# The effect of a training program on maternal role adaptation and self-esteem of mothers with preterm infants: a quasi-experimental study

**DOI:** 10.1186/s12905-021-01440-z

**Published:** 2021-08-11

**Authors:** Maryam Sohrabi, Mansooreh Azizzadeh forouzi, Roghayeh Mehdipour-Rabori, Behnaz Bagherian, Monirsadat Nematollahi

**Affiliations:** 1 Department of Pediatrics and Neonatal Intensive Nursing, Razi Faculty of Nursing and Midwifery, Kerman University of Medical Science, Kerman, Iran; 2 Nursing Research Center, Kerman University of Medical Sciences, Kerman, Iran

**Keywords:** Maternal role adaptation, Self-esteem, Preterm infants, Training programs, Neonatal intensive care unit

## Abstract

**Background:**

Admission of preterm infants in the neonatal intensive care unit limits the mother’s interaction with their infants, delaying accepting and playing the motherhood role. Besides, mothers of preterm infants have low self-esteem due to their infants' condition. Accordingly, the present study explored the effect of implementing the training program on maternal role adaptation and self-esteem of mothers of preterm infants admitted to the neonatal intensive care unit.

**Methods:**

This study employed a quasi-experimental design with two groups. The participants were 80 mothers of preterm infants. The participants were selected using convenience sampling and simply randomly assigned to the intervention and control groups. The instruments included a demographic information questionnaire, the Rosenberg Self-Esteem Scale, and the Maternal Role Adaptation Scale. The participants in the intervention group attended the training program, while the control group did not receive any intervention. The questionnaires were completed by the two groups before and 2 weeks after the intervention. The collected data were analyzed using SPSS software version 21, a significance level of 0.05.

**Results:**

The maternal role adaptation scores before the intervention in the control and intervention groups were 134.222 ± 11.84 and 138.800 ± 12.42, respectively, showing no statistically significant difference (P = 0.096). The corresponding scores after the intervention for the control and intervention groups were 139.17 ± 12.46 and 154.05 ± 8.57, showing a significant intergroup difference (P < 0.001). Similarly, the pre-intervention self-esteem scores in the control and intervention groups were 30.30 ± 3.79 and 30.95 ± 8.61, showing no statistically significant difference between the two groups (P = 0.664). Besides, the post-intervention self-esteem scores in the control and intervention groups were 31.52 ± 3.42 and 36.001 ± 7.74, respectively, indicating a statistically significant difference between the two groups (P < 0.001).

**Conclusion:**

Given the insight from this study, implementing training programs is a suitable solution for improving maternal role adaptation and increasing mothers' self-esteem. Furthermore, nurses’ training packages can help the mother accept the maternal role more quickly and improve the mother's self-esteem for better care of the baby.

*Trial registration* The registration number for this study was obtained from Kerman University of Medical Sciences, and the number of the grant was 98000150.

## Background

Prematurity is the most common cause of neonatal hospitalization. According to the World Health Organization, infants whose fetal age is less than 37 weeks at birth are called preterm infants [1]. Although the frequency of preterm labor varies considerably between countries, almost 90% of these premature births occur in developing countries in Africa and Asia. In 2014, the rate of preterm births was 10% in the US, while in Europe in 2010, preterm birth rates varied markedly from 5 to 10.6% among live births [2].

Preterm delivery plays a major role in developing LBW. A systematic review and meta-analysis reported the prevalence of preterm labor to be 9.2% in Iran [3].

The mother is the infant’s primary caregiver, and motherhood is the most critical role of a woman [4]. Besides, adaptation to the maternal role involves conceptualizing and establishing motherhood, which is characterized by forming a new identity and maternal behaviors [5]. However, motherhood for a baby admitted to the neonatal intensive care unit (NICU) is very different from motherhood for a full-term baby taken home at the earliest opportunity after birth [4]. A mother can take care of her baby once she accepts her role as a mother [6]. In contrast, mothers who cannot adapt to their role have low performance in their role, especially in their child's attachment [7]. According to Mercer's theory of motherhood, an infant's mother admitted to the neonatal intensive care unit has a limited opportunity to care for and interact with the infant, which delays accepting and playing the maternal role [5]. Factors such as age, maternal self-esteem, education, number of pregnancies, anxiety, depression, marital status, maternal personality traits, delivery experience, infant health status, social support from the husband and family members, and health and medical staff affect maternal role [8]. The most important reason for not accepting the maternal role is the mother’s unpreparedness to become a mother during pregnancy [9]. The birth of preterm infants disturb the balance in the activities and roles of the mother, leads to a feeling of inadequacy in the mother in caring for the infant and lowers her self-esteem [10].

According to Rosenberg, self-esteem refers to the feeling of self-sufficiency to face fundamental challenges in life. Self-esteem stems from the difference between the ideal and perceived self, and the more significant difference between the two, the lower the self-esteem [11]. Kou et al. (2012) showed that maternal self-esteem facilitates the development of maternal roles such as breastfeeding and also promotes the growth of the child [12]. Mothers who lack self-esteem in the postpartum period may not adequately care for their infants [13, 14]. Preparing a pregnant woman to accept a mother's role is one of the essential responsibilities of nurses. However, in Iran, most of the pregnancy measures include the mother's physical care, and less attention is paid to the mother's psychological needs [15]. Furthermore, as caring for the immature infant engages the family, especially the mother [10], providing education for mothers about their motherly role is essential [16]. Providing information about baby care to the family will make them feel more in control of their situation, develop a more realistic view of the baby, and participate more in the infant's care. Engaging the family members in infant care can improve the parents ‘ability to care for infants during and after admission and reduce the readmission of infants [17]. Adaptation to the maternal role also develops when the mother has the knowledge and skills of caring for the baby required for playing the maternal role [18]. Nurses can minimize mothers’ mental status by designing and implementing training programs focusing on the maternal role [19]. Training programs are cost-effective, and their implementation does not require modern equipment [20]. Providing purposeful information and training and practicing activities related to parenting can reduce anxiety and increase confidence in the mother and increase the level of communication between mother and baby [21]. Given the issues highlighted above and the researcher's experience, mothers with preterm infants do not have the opportunity to accept and adapt to the maternal role due to the physical problems of infants during hospitalization.

Moreover, due to the separation from their baby, these mothers share their motherly role with nurses and doctors, and in many cases, they cannot be with their child for a long time, and this disturbs adapting to their motherly role. Furthermore, these mothers suffer from low self-esteem due to their baby's care sensitivities. They have doubts about their ability to care for preterm infants, which can affect the quality of their infant care. Given that nurses in neonatal intensive care units in Iran do not provide structured training programs for mothers of preterm infants, this study aims to explore the effect of implementing the training program on maternal role adaptation and the self-esteem of mothers of preterm infants admitted to the neonatal intensive care unit of hospitals in Kerman in 2020.

The hypothesis of this study was “implementing the training program effect on maternal role adaptation and the self-esteem of mothers with preterm infants.

## Methods

This study employed a quasi-experimental design with two groups and pre-test and post-test. The study was conducted in neonatal intensive care units in Afzalipour Hospital in Kerman. Afzalipour Hospital has 96 beds for infants' hospitalization in the intensive care unit and has all the facilities and equipment required for the care of preterm infants and is the largest educational and medical center in southeastern Iran.

### Sampling method

Reviewing the literature [22] and considering the power as 80%, (β = 20%) and α = 0.05, the sample size was estimated 74 mothers in the intervention and the control groups (totally 74 samples)by Koopak formula using G*Power version 3.1.9.7. However, regarding the probability of dropouts and an increase in the power of our study, 80 samples were assigned in the intervention and control groups, respectively (total 80 samples). All of the participants completed the study.

The inclusion criteria were the mothers with preterm infants under 37 weeks of gestation, having the ability to speak Persian, being married, having no physical and mental problems, and having no infants with congenital anomalies and other diseases. Mothers whose children have congenital anomalies, and individuals with mental disorders (based on self-report), individuals who were engaging in substance abuse, or who had experienced individual or family crisis in the previous 6 months were excluded.

Then, to do the randomization process, first, a list of the admitted neonates who met the inclusion criteria was prepared and they were divided into control and intervention groups by simple random sampling using a random number table.

### Data collection

The instruments to collect the data were the demographic characteristics of mothers and infants, the Rosenberg Self-Esteem Scale, and the Maternal Role Adaptation Scale.

### Demographic characteristics questionnaire

The characteristic demographic questionnaire for mothers assessed the mothers’ age, level of education, income status, marital status, having information about neonatal care, Informational sources of preterm neonatal care, children number, stillbirth number, live birth number, pregnancy number, delivery type, pregnancy age. Also, demographic characteristics for infants included birth weight, current weight, admission days, being under mechanical ventilation.

### The Rosenberg Self-Esteem Scale

The Rosenberg Self-Esteem Scale is a 10-item tool measuring a person’s positive and negative feelings about himself/herself. Each item is scored on a four-point scale (strongly agree, agree, disagree, and strongly disagree). The total score on the scale ranges from 10 to 40. Five items (items 1 to 5) are worded positively (4 = strongly agree, 3 = agree, 2 = disagree, and 1 = strongly disagree) and the remaining 5 items (items 6 to 10) are worded negatively and are scored reversely (1 = strongly agree, 2 = agree, 3 = disagree, and 4 = strongly disagree). A higher score on this scale indicates the respondents’ higher self-esteem. Besides, the Cronbach's alpha coefficient for this scale was calculated to be 0.74, implying its acceptable reliability [23].

### The Maternal Role Adaptation Scale

The Maternal Role Adaptation Scale is a 32-item that contains six subscales (subthemes): (1) Participation in care, (2) Self-efficacy, (3) Distant mothering, (4) Uncertainty, (5) Interaction, and (6) Growth and development. The scale was developed by Haidarpour et al. (2016) for use in Iran. This instrument’s validity was evaluated and confirmed using face and content validity, and its reliability was measured using internal consistency and the test–retest method. The Cronbach's alpha and the intra correlation coefficient (ICC) were 0.77 and 0.18, respectively. The items in the instrument are scored using a five-point Likert scale (5 = strongly agree, 4 = agree, 3 = undecided, 2 = disagree, and 1 = strongly disagree). The total score on the scale ranges from 32 to 160, and a higher score indicates the mother’s better adaptation to the maternal role. Items 7, 11, 12, and 13 are scored reversely (1 = strongly agree, 2 = agree, 3 = undecided, 4 = disagree, and 5 = strongly disagree) [24].

### Data analysis

The collected data were analyzed using SPSS software (version 21). Descriptive statistics (frequency, percentage, mean, and standard deviation) were used to describe the participants’ demographic characteristics. Besides, descriptive indices (mean and standard deviation) and the independent samples t-test were used to compare the mean scores of self-esteem and maternal role adaption between the two groups before and after the intervention. Finally, the paired samples t-test was used to compare maternal role adaption and self-esteem before and after the intervention in each group.

### Intervention

The study population was mothers of preterm infants that were born during 2020 and met inclusion criteria. Also, the time of enrollment mothers was upon admission to NICU.

Before the intervention, the three questionnaires (demographic information, the Rosenberg Self-Esteem Scale, and the Maternal Role Adaptation Scale) were completed by the participants in two groups.

To implement the intervention for the mothers of the intervention group, the researcher provided some instructions and training based on the items in the questionnaires. In the first session the characteristics of preterm infants, the importance of breastfeeding, correct breastfeeding, embracing the baby were trained. In the second session, the mothers were asked to apply the trained care tips to the doll that was given to them. The researcher observed how the mothers performed in the instructed items. If the mother had any problems or made any mistakes, the researcher helped them to perform the procedures correctly. In the third session Hand expression and correct pumping, and the fourth session post-discharge follow-ups including ear and eye control, baby vaccinations, thyroid testing, and breastfeeding retraining were thought and performed by the mothers. The researcher observed the procedure and intervened if necessary. The training program was held during 4 one-hour sessions for four consecutive days in the waiting room of the neonatal intensive care unit. To give feedback on the training program's efficiency, the researcher trained the mothers in groups of four. The three questionnaires were completed again by the participants in both groups 2 weeks after the intervention.

The members of the control group received routine training, and they did not receive a planned and organized training program with getting feedback and following them for doing correctly. In addition, the mothers in the control group did not do any practice on the doll with supervising their nurse before taking care of their infants.

Besides, after the intervention, an educational booklet containing the instruction content of the training program was given to the participants in the control group. Also, in this study, the participants and educators were not blinded. To ensure the participants in the two groups not to be in contact and not to exchange information, the members of the two groups were selected from separate work shifts. For example, if sampling was done from the intervention group in the Saturday morning shift, sampling from the control group would be done on other days. Besides, the training program was held for the participants in the intervention group in a separate room where the members of the control group were not present.

### Ethical consideration

The present research was conducted after taking the code of ethics (IR.KMU.REC. 1398.164) from the ethics committee of Kerman University of Medical Sciences. The researcher explained the research aims and method to nurses of the neonatal intensive care units and infants’ mothers. The infants’ families were ensured of information confidentiality, and they could be aware of research results with a phone contact. The infants’ mothers were ensured that they could participate in the study voluntarily and were free to withdraw from the study. Written consents were taken from infants’ mothers.

## Results

The results showed that there was no statistically significant difference between the two groups in terms of demographic characteristics of the mothers and their preterm infants, and the two groups were comparable in terms of the demographic characteristics (P > 0.05) (Tables 1, 2).

**Table 1:** Demographic characteristics of mothers of preterm infants of two groups (n = 80)

Group	Intervention (n = 40)		Control (n = 40)		P-value
Demographic characteristics	Number	Percent	Number	Percent	
Age (year)**					P = 0.06*** F (1.78) = 483.4
< 25	3	7.5	8	20	
25–30	14	35	17	42.5	
> 30	23	57.5	15	37.5	
Mean ± SD	31.58 ± 5.81	28.82 ± 5.80			
Education level***					
Diploma/middle/high school	26	65	27	67.5	P = 0.549*** F = 1.354
Bachelor’s/associate	13	32.5	10	25	
Master’s/higher	1	2.5	3	7.3	
Income (tomans)					P = 0.160*** F = 3.462
< 1.500000	20	50	15	37.5	
1.500000– 3.500000	18	45	25	62.5	
> 3.500000	2	5	0	0	
Marital status***					P = 1*** F = 1.001
Single (widow/or, divorced)	1	2.5	0	0	
Married	39	97.5	40	100	
Having information about neonatal care*					P = 1* χ^2^ = 0
Yes	11	27.5	11	27.5	
No	29	72.5	29	72.5	
Informational sources of preterm neonatal care***					P = 0.670*** F = 2.968
Healthcare providers	6	15	8	20	
Book	3	7.5	3	7.5	
Internet	2	5	0	0	
Others	1	2.5	0	0	
None	28	70	29	72.5	
Children no.*					P = 0.540* χ^2^ = 2.182
1	10	25	12	30	
2	12	30	16	40	
3	12	30	9	22.5	
> 3	6	15	3	7.5	
Live birth no.*					P = 0.075* χ^2^ = 5.328
1	10	25	15	37.5	
2	15	37.5	19	47.5	
≥ 3	15	37.5	6	15	
Stillbirth no.***					P = 0.768 Fisher’s exact test = 0.755
0	27	67.5	28	70	
1	9	22.5	10	25	
≥ 2	4	10	2	5	
Pregnancy no.*					P = 0.153* χ^2^ = 3.750
1	9	22.5	10	25	
2	10	25	17	42.5	
≥ 3	21	52.5	13	32.5	
Delivery type*					P = 0.179 χ^2^ = 1.805*
Vaginal	18	45	24	60	
Cesarean	22	55	16	40	
Gestational age (week)****					P = 0.539**** Z = 0.740
≤ 34	16	40	11	27.5	
> 34	24	60	29	72.5	
Mean ± SD	34.52 ± 1.86		34.32 ± 2.94		
Max–Min	30–36		26–36		

*Chi-square test

**Analysis of variance test

***Fisher’s exact test

****Mann–Whitney U test

**Table 2:** Demographic characteristics of admitted preterm infants in the study groups (n = 80)

Group	Intervention (n = 40)		Control (n = 40)		P-value
Demographic characteristics	Number	Percent	Number	Percent	
Birth weight (g)*					P = 0.278* Z = 0.687
≤ 2200	16	40	11	27.5	
> 2200	24	60	29	72.5	
Mean ± SD	2308.38 ± 507.90	2375.50 ± 625.49			
Current weight (g)*					P = 0.230* Z = 0.675
≤ 2200	17	42.5	11	27.5	
> 2200	23	57.5	29	72.5	
Mean ± SD	2299.12 ± 508.69	2388.87 ± 640.36			
Duration of hospitalization *					P = 0.440* Z = 0.721
≤ 5	30	75	31	77.5	
> 5	10	25	9	22.5	
Mean ± SD	4.53 ± 3.47	4.85 ± 3.47			
Being under Mechanical ventilation**					P = 0.785** χ^2^ = 0.075
Yes	8	20	9	22.5	
No	32	80	31	77.5	

*Mann–Whitney U

**Chi-square

The results of the paired samples t-test suggested that the mean scores of maternal role adaptation and its subscales showed a statistically significant difference before and 2 weeks after the intervention in the intervention group (P < 0.001), implying the maternal role training for the mothers of preterm infants hospitalized in the neonatal intensive care unit increased the maternal role adaptation and its subscales compared to the pre-intervention stage (Table 4). Besides, the results of the paired samples t-test showed that the mean scores of maternal role adaptation and its subscales were statistically significant in the control group before and 2 weeks after the intervention (P < 0.001), indicating the scores of maternal role adaptation and its subscales increased 2 weeks after the intervention compared to before the intervention and this increase was significant (P < 0.05) (Table 3). Also, the results of the independent t-test showed that the changes in the mean scores of mother role adaptation, in all dimensions except the interaction dimension, were a significant difference between the two groups (Table 5).

**Table 3:** Mean scores of maternal role adaptation and its dimensions in control and intervention groups

Maternal role adaptation and its dimensions	Group	Before intervention	After intervention	Within-group estimate
Involvement in care	Intervention	62.97 ± 7.20	68.50 ± 2.93	P < 0.001* T = − 6.315
	Control	59.37 ± 5.92	61.45 ± 6.139	P < 0.001* T = − 3.967
	Between-group estimate	P = 0.017** T = 2.441	P < 0.001** T = 44.014	
Self-efficacy	Intervention	26.45 ± 2.60	28.90 ± 1.29	P < 0.001* T = − 8.240
	Control	25.87 ± 2.74	26.67 ± 2.70	P = 0.004* T = − 3.035
	Between-group estimate	P = 0.339** T = 0.961	P < 0.001** T = 4.695	
Distant motherhood	Intervention	12.80 ± 1.58	14.42 ± 0.873	P < 0.001* T = − 8.193
	Control	12.67 ± 1.52	12.95 ± 1.61	P = 0.020* T = -2.430
	Between-group estimate	T = 0.359 P = 0.721**	T = 5.077 P < 0.001**	
Lack of trust	Intervention	14.95 ± 3.07	17.72 ± 2.21	P < 0.001* T = − 6.864
	Control	15.15 ± 3.27	15.92 ± 2.99	P < 0.001* T = − 3.583
	Between-group estimate	P = 0.779 T = -0.282	P = 0.003 T = 3.057	
Interaction	Intervention	12.55 ± 2.15	14.80 ± 6.40	P = 0.029* T = − 2.269
	Control	12.42 ± 1.55	13.17 ± 1.44	P < 0.001* T = − 4.713
	Between-group estimate	P = 0.767** T = 0.297	P = 0.122** T = 1.564	
Growth	Intervention	9.07 ± 0.97	9.70 ± 0.46	P < 0.001* T = − 4.900
	Control	8.72 ± 1.03	9.001 ± 1.06	P = 0.010* T = − 2.718
	Between-group estimate	P = 0.123** T = 1.558	P < 0.001** T = 3.819	
Maternal role adaptation	Intervention	138.800 ± 12.42	154.05 ± 8.57	P < 0.001* T = − 9.437
	Control	134.222 ± 11.84	139.17 ± 12.46	P < 0.001* T = − 4.900
	Between-group estimate	P = 0.096** T = 1.685	P < 0.001** T = 6.219	

*Paired t-test

**Independent t-test

**Table 4:** Mean score of maternal self-esteem in control and intervention groups

Maternal self-esteem and its dimensions	Group	Before intervention	Two weeks after intervention	Within-group estimate
Personal competency	Intervention	18.35 ± 7.97	21.37 ± 6.96	P = 0.024* T = -2.243
	Control	17.97 ± 2.76	18.82 ± 2.37	P < 0.001* T = − 3.479
	Between-group estimate	P = 0.780** T = 0.281	P = 0.031** T = 2.193	
Self-satisfaction	Intervention	12.60 ± 1.49	14.62 ± 1.67	P < 0.001* T = − 7.733
	Control	12.32 ± 1.43	12.70 ± 1.52	P = 0.014* T = − 2.564
	Between-group estimate	P = 0.405** T = 0.837	P < 0.001** T = 5.379	
Maternal self-esteem	Intervention	30.95 ± 8.61	36.001 ± 7.74	P = 0.002* T = − 3.245
	Control	30.30 ± 3.79	31.52 ± 3.42	P < 0.001* T = − 3.837
	Between-group estimate	P = 0.664** T = 0.437	P < 0.001** T = 3.343	

*Paired t-test

**Independent t-test

**Table 5:** Numerical indices of differences in adaptation of maternal role and self-esteem and its dimensions in mothers between two groups

Variables	Intervention group	Control group	P-value
	Mean ± SD	Mean ± S D	
Involvement in care	5.52 ± 5.53	2.07 ± 3.30	P < 0.001* T = 3.385
Self efficacy	2.54 ±1.58	0.08 ± 1.66	P < 0.001* T = 4.153
Distant mothrhood	1.62 ± 1.25	0.27 ± 0.71	P < 0.001* T = 5.91
Lack of trust	2.77 ± 2.55	0.77 ± 1.36	P < 0.001* T = 4.362
Interaction	2.25 ± 6.27	0.75 ± 1.01	P = 0.139* T = 1.494
Growth	0.62 ± 0.80	0.27 ± 0.64	P = 0.035* T = 2.150
Maternal role adaptation	15.25 ± 10.22	4.95 ± 6.38	P < 0.001* T = 5.405
Personal competency	3.02 ± 9.79	0.85 ± 1.54	P = 0.010* T = 2.455
Self-satisfaction	2.02 ± 1.65	0.37 ± 0.92	P < 0.001* T = 5.501
Maternal self-esteem	5.05 ± 9.84	1.22 ± 2.01	P = 0.018* T = 2.408

*T-independent test

Before the intervention, the intervention and control groups were 134.222 ± 11.84 and 138.800 ± 12.42, respectively, showing no statistically significant difference as indicated by the independent samples t-test (P = 0.096). Besides, corresponding scores after the intervention for the control and intervention groups were 139.17 ± 12.46 and 154.05 ± 8.57, showing a significant intergroup difference (P < 0.001) (Table 3).

Furthermore, the mean scores of participation in care before the intervention for the participants in the intervention and control groups were 62.97 ± 7.20 and 59.37 ± 5.92, respectively, showing statistically significant intergroup differences before the intervention as indicated by the independent samples t-test (P = 0.017) in favor of the intervention group. Besides, mean scores of participation in care after the intervention for the participants in the control group (61.45 ± 61.139) and the intervention group (68.50 ± 2.39) showed a statistically significant difference (P < 0.001).

The self-efficacy scores before the intervention for the participants in the intervention and control groups were 26.45 ± 2.60 and 25.87 ± 2.74, respectively, showing no statistically significant difference as indicated by the independent samples t-test (P = 0.339) and the two groups were comparable in terms of self-efficacy before the intervention. Moreover, corresponding scores for self-efficacy after the intervention for the participants in the intervention and control groups were 28.90 ± 1.29 and 26.67 ± 2.70, implying a significant intergroup difference in favor of the intervention group (P < 0.001). Similarly, the distance mothering scores before the intervention for the participants in the intervention and control groups were 12.80 ± 1.58 and 12.67 ± 1.52, respectively, showing no statistically significant difference (P = 0.721). However, the corresponding scores for distant mothering after the intervention for the participants in the intervention and control groups were 14.42 ± 0.873 and 12.95 ± 1.63, implying a significant intergroup difference in favor of the intervention group (P < 0.001).

The study results suggested that the uncertainty scores before the intervention for the intervention and control groups were 14.95 ± 3.07 and 15.15 ± 3.27, respectively, showing no significant difference between the two groups (P = 0.779). In contrast, the corresponding post-intervention uncertainty scores reported by the intervention and control groups were 17.72 ± 2.21 and 15.92 ± 2.99, indicating a significant intergroup difference in favor of the intervention group (P < 0.003).

The pre-intervention interaction scores reported by the participants in the intervention and control groups were 12.55 ± 2.15 and 12.42 ± 1.55, respectively, showing no significant difference between the two groups as suggested by the independent samples t-test (P = 0.767). The corresponding post-intervention scores reported by the participants in the two groups were 14.80 ± 6.40 and 13.17 ± 1.44, indicating no significant intergroup difference (P < 0.122).

It was also shown that the development and growth scores before the intervention for the intervention and control groups were 9.07 ± 0.97 and 8.72 ± 1.03, respectively, showing no significant difference between the two groups (P = 0.123). However, the corresponding post-intervention scores on self-reported development and growth of the participants in the intervention and control groups were 9.70 ± 0.46 and 9.001 ± 1.06, demonstrating a significant intergroup difference in favor of the participants in the intervention group (P < 0.001) (Table 3).

The results of the study also suggested that mean scores of the participants' self-esteem and its subscales before and 2 weeks after the intervention in the intervention group were significantly different (P < 0.001), suggesting that implementing the training program for the mothers of preterm infants admitted to the neonatal ward increased the mothers' self-esteem and its subscales compared to the pre-intervention period. Besides, the results of the paired samples t-test showed that the mean scores of self-esteem and its subscale before and 2 weeks after the intervention were statistically significant for the participants in the control group (P < 0.001) (Table 4). Also, the results of the independent t-test showed that the changes in the mean scores of mother self -esteem, in all dimensions, was a significant difference between the two groups (Table 5).

The self-esteem scores before the intervention for participants in the intervention and control groups were 30.95 ± 8.61 and 30.30 ± 3.79, respectively, showing no significant difference between the two groups (P = 0.664).

Besides, the mean scores of personal competence as a dimension of self-esteem for the intervention and control groups were 18.35 ± 7.97 and 17.97 ± 2.76, respectively, showing no significant difference between the two groups (P = 0.780). However, the corresponding post-intervention scores on personal competence for the participants in the control and intervention groups were 31.52 ± 3.42 and 36.001 ± 7.47, showing a significant intergroup difference in favor of the participants in the intervention group (P < 0.001). Finally, the mean scores of self-satisfaction for the participants in the control and intervention groups were 12.32 ± 1.43 and 12.60 ± 1.49, respectively, showing no significant difference (P = 0.405). In contrast, the corresponding post-intervention scores on self-satisfaction for the participants in the control and intervention groups were 12.70 ± 1.52 and 14.62 ± 1.67, showing a significant intergroup difference in favor of the participants in the intervention group (P < 0.001) (Table 4).

## Discussion

The results of the present study showed that the mean scores of maternal role adaptation for the participants in the two groups before the intervention were not significantly different, and the two groups were identical. However, the mean scores of maternal role adaptation for the intervention group participants were higher compared to the control group, and the two groups were significantly different in terms of adaptation to the maternal role. It should be noted that all subscales of maternal role adaptation, including participation in care, self-efficacy, distant mothering, uncertainty, interaction, and development and growth were significantly different after the intervention compared to the pre-intervention stage.

The maternal role adaption is one of the most critical factors in performing care tasks by the mother. Bailey emphasized that a mother can take care of her baby when she has accepted her role as a mother [25]. However, mothers of preterm infants face completely different conditions compared to mothers of full-term infants without physical problems.

In their study, Bialoskurski et al. showed that the unreliable condition of a preterm infant delays the mother-infant relationship [26]. Early initiation of nursing interventions to facilitate parents’ communication, especially mothers’ communication with their hospitalized baby, has a significant impact on establishing effective parental-infant communication since ineffective and cold communication is challenging to change [27]. Moreover, Johnson showed that parental support is one of the most important tasks of the health care team, especially nurses [28], and preparing mothers to adapt to the maternal role is one of these tasks because the more prepared mothers are, the more quality care they can provide [29]. Mothers can be prepared to assume their roles through psychotherapy training activities or familiarity with maternal roles.

The results of a study by Pouyan et al. (2019) suggested that the effect of interpersonal psychotherapy training on stress and adaptation to the maternal role for mothers with preterm delivery reduced maternal stress score and resulted in a better adaptation of mothers [30]. Similarly, the present study supported the positive effects of training for the mothers of preterm infants. However, the mentioned study's training was based on interpersonal psychotherapy, which was different from the current study. Kurdi et al. also showed that the maternal role training program held for nulliparous women with unplanned pregnancies led to an improvement in the maternal role of the women under study [31]. In a similar vein, the present study highlighted the effectiveness of maternal role training on mothers’ competence. However, in the study conducted by Kurdi et al., the long-term intervention was provided during pregnancy and postpartum. In another study, Baghdari et al. (2016) observed that the conformity of pregnancy and acceptance of a maternal role in the pregnant woman was improved using a training intervention [32]. The authors used the mother-fetus attachment scale. A study by Heidari et al. (2019) also showed the effectiveness of a breastfeeding self-efficacy training program in nulliparous women [33]. The authors used distance education as a training technique.

Ngai et al. (2009) stated that childbirth psychology training had no effect on maternal role competence immediately and 6 weeks after childbirth [34], perhaps one of the reasons for the ineffectiveness of the training program in this study was that pregnant women did not have adequate information about their maternal role, while the mothers of preterm infants in the present study had developed their maternal roles.

Gao et al. (2012) showed that the maternal role training program did not affect maternal role competence in 6 weeks postpartum but it was effective after 3 months [35], highlighting a long-term effect OF training programs. Heydarpour et al. (2017) also found that effective adaptation to the maternal role increases mothers' confidence and satisfaction in caring for their children [36]. Similarly, the present study showed that the implementation of the training program caused a significant increase in the self-esteem of the mothers in the intervention group, while there was no increase in the self-esteem score of the mothers who did not receive the intervention. It was also shown that the self-esteem subscales, including personal competence and self-satisfaction, improved significantly after the intervention for the mothers in the intervention group. The birth of premature infants disrupts family activities and plans. It leads to inadequacy in the mother in caring for the baby and reduces the mother's self-esteem [37]. Aklechi and Mehri (2012) showed that low self-esteem causes feelings of inadequacy, loneliness, and decreased performance [38]. Kou et al. (2012) also found that promoting maternal self-esteem facilitates maternal roles such as breastfeeding and promotes child growth [12]. Mothers who lack self-esteem in the postpartum period may not be able to care for their infants adequately and may not be successful in breastfeeding [13, 14]. The present study showed that providing training on how to care for the child increases mothers' self-esteem. In contrast, Arzani et al. (2012) showed that hugging maternal care training did not affect the self-esteem of mothers of premature infants but increased the feeling of independence and self-confidence and led to greater satisfaction of mothers in playing their maternal role [22]. Perhaps one of the reasons for the mothers' low self-esteem in this study was the small number of participants that can be regarded as one of the limitations of this study.

Mc Carter and Perez-Blasco showed that mothers with low self-esteem could not adequately care for their infants and did not breastfeed successfully [13, 14]. Amel Barez et al. (2015) showed that self-esteem affects mothers' self-efficacy, feelings, and performance [39]. They suggested that the care team should pay attention to the psychosocial aspects that affect mothers' performance, including self-esteem [39]. In another study, Nafariye et al. (2020) reported the effectiveness of mindfulness training on self-esteem and emotion regulation of mothers of children with learning disabilities [40]. Although the type of intervention was cognitive-behavioral counseling in this study, it improved the mothers' self-esteem.

Furthermore, educational interventions with psychological and supportive approaches were shown to positively affect the self-esteem of poorly supervised adolescents [41] and adolescents with cerebral palsy [42]. Aghajani et al. (2018) highlighted the positive effects of life skills training on self-esteem and the psychological distress of addicted women [43]. Although the research sample and the type of training were utterly different from the current study, the training program's implementation and acquisition of more life skills improved the participants’ self-esteem.

## Limitation and implications

One of the limitations of this study, which was not pure Randomized Control Trial, and it affects the outcomes of this study. Besides, the infants’ restlessness and the mothers’ fatigue and impatience could negatively affect the mothers’ responses and their motivation to participate in the study. To overcome this limitation, the researcher tried to provide the questionnaire to the parents when they had enough time and patience as well as when they were mentally prepared. If a mother was absent in a training session, she received the instructions for the missing session individually. Also, team researcher suggested that nurses in NICU implement training programs for improving maternal role adaptation and increasing mothers' self-esteem. Because of nurses’ training packages can help the mother accept the maternal role more quickly and improve the mother's self-esteem for better care of the baby.

## Conclusion

The present study showed that maternal role training improved the maternal role adaptability and self-esteem of the mothers of preterm infants. Providing instructions on how to care for infants can be an effective intervention technique to make mothers of preterm infants more adaptable to their maternal role and increase their self-esteem, thus improving the quality of maternal care and the condition of infants. Given the paucity of research in this field, there is a need for more training interventions to come up with more reliable and accurate results.

## Data Availability

The datasets used and/or analyzed during the current study are available from the corresponding author on reasonable request.
